# Do scoring systems predict mortality following emergency abdominal aortic aneurysm repair? The Norwich experience

**DOI:** 10.1186/cc9924

**Published:** 2011-03-11

**Authors:** S Kumar, J Nortje

**Affiliations:** 1 Norfolk & Norwich University Hospital, Norwich, UK

## Introduction

APACHE II scores [1] and Glasgow Aneurysm Scores (GAS) [2] are commonly used in ICUs to predict mortality. These scoring systems (scores α mortality), when applied to postoperative emergency open abdominal aortic aneurysm (AAA) repair patients, yield varying results. We applied these scoring systems to our patients to establish their predictive value in our clinical setting.

## Methods

This retrospective audit included patients who underwent emergency open AAA repair and were admitted to our ICU, over a period of 1 year (November 2008 to November 2009). These patients were identified from our local ICU database (Metavision^®^) and scores (APACHE II and GAS) were calculated for each of these patients. The mortality rates were compared with the national average [3].

## Results

**Figure 1:**
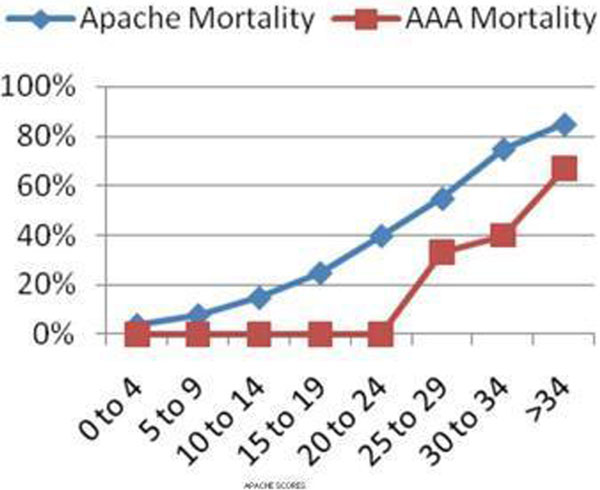
**AAA mortality versus APACHE mortality**.

**Figure 2:**
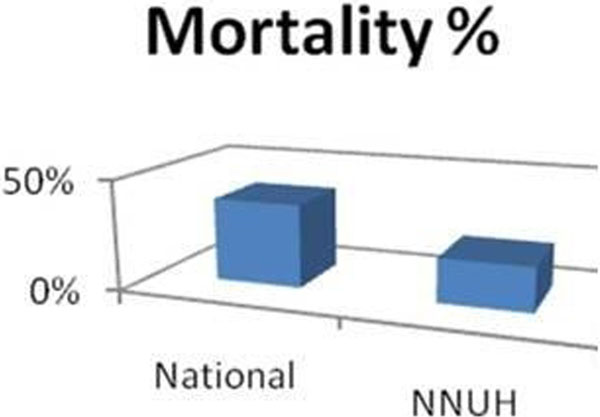
**AAA mortality NNUH versus UK**.

A total of 98 AAA repair patients were identified, of whom 35 patients (32 males and three females) had undergone emergency (ruptured) repair. Seven patients (20%), including two females, died in the ICU. There is an increase in mortality with increasing APACHE II scores (Figure 1). The same does not apply for GAS scores but all the patients who died had a GAS score >89. Our mortality rate was 20% compared with the national mortality of 38% (Figure 2).

## Conclusions

APACHE II scores seem to be more predictive of our unit AAA mortality rates than GAS scores. We aim to apply these scores to a larger dataset and also determine possible reasons for improved survival.
